# Awareness of basic life support among medical, dental, nursing students and doctors

**DOI:** 10.4103/0019-5049.63650

**Published:** 2010

**Authors:** Shanta Chandrasekaran, Sathish Kumar, Shamim Ahamed Bhat, Saravanakumar, P Mohammed Shabbir, VP Chandrasekaran

**Affiliations:** 1 Vinayaka Mission's Kirupananda Variyar Medical College, Salem, Tamilnadu, India; Department of Accident and Emergency, Vinayaka Mission's Kirupananda Variyar Medical College, Salem, Tamilnadu, India

**Keywords:** BLS awareness, AED, CPR questionnaire

## Abstract

To study the awareness of Basic Life Support (BLS) among students, doctors and nurses of medical, dental, homeopathy and nursing colleges. A cross-sectional study was conducted by assessing responses to 20 selected basic questions regarding BLS among students, doctors and nurses of medical, dental, homeopathy and nursing colleges. After excluding the incomplete response forms the data was analysed on 1,054 responders. The results were analysed using an answer key prepared with the use of the Advanced Cardiac Life Support manual. Out of 1,054 responders 345 were medical students, 75 were medical interns, 19 were dental students, 59 were dental interns, 105 were homeopathy interns, 319 were nursing students, 72 were doctors, 29 were dentists, 25 were nursing faculty and six were homeopathy doctors. No one among them had complete knowledge of BLS. Only two out of 1054 (0.19%) had secured 80 – 89% marks, 10 out of 1054 (0.95%) had secured 70 – 79% marks, 40 of 1054 (4.08%) had secured 60 – 69% marks and 105 of 1054 (9.96%) had secured 50 – 59% marks. A majority of them, that is, 894 (84.82%) had secured less than 50% marks. Awareness of BLS among students, doctors and nurses of medical, dental, homeopathy and nursing colleges is very poor.

## INTRODUCTION

Basic Life Support (BLS) includes recognition of signs of sudden cardiac arrest (SCA), heart attack, stroke and foreign-body airway obstruction (FBAO); cardiopulmonary resuscitation (CPR); and defibrillation with an automated external defibrillator (AED).[1] It is very important that every person in the community know about Basic Life Support to save lives and improve the quality of community health. At least the doctors, nursing and paramedical staff are expected to know about it, as they are frequently facing life threatening situations and the knowledge of BLS will be definitely useful. In this study we wanted to investigate the awareness of Basic Life Support among various health sector persons.

### Aim

To study the awareness of Basic Life Support (BLS) among students, doctors and nurses of medical, dental, homeopathy and nursing colleges.

## METHODS

A cross-sectional study was conducted by assessing the responses to 20 selected basic questions regarding BLS, among students, doctors and nurses of the medical, dental, homeopathy and nursing colleges in a city in Tamilnadu, India.

A questionnaire with 20 questions regarding the awareness and skills involved in BLS was used to assess the levels of awareness to BLS and its practical knowledge. The aspects on which they were interrogated were about the abbreviation of BLS, AED and EMS (Emergency Medical Service), sequential steps in BLS, assessment and resuscitation techniques with regard to airway, breathing, circulation in unresponsive victims of different age groups, techniques regarding removal of foreign body obstruction, recognition of early signs of stroke and acute coronary syndrome [Table 1].

**Table 1:** Questionnaire

What is the abbreviation of “BLS”? Best Life Support Basic Life Support Basic Lung Support Basic Life Services When you find someone unresponsive in the middle of the road, what will be your first response? (Note: You are alone there) Open airway Start chest compression Look for safety Give two breathings If you confirm somebody is not responding to you even after shaking and shouting at him, what will be your immediate action? Start CPR Activate EMS Put him in recovery position Observe What is the location for chest compression? Left side of the chest Right side of the chest Mid chest Xiphisternum What is the location for chest compression in infants? One finger breadth below the nipple line One finger breadth above the nipple line At the intermammary line At Xiphisternum If you do not want to give mouth-to-mouth CPR, the following can be done *EXCEPT* Mouth-mask ventilation and chest compression Chest compression only Bag mask ventilation with chest compression No CPR How do you give rescue breathing in infants? Mouth-to-mouth with nose pinched Mouth-to-mouth and nose Mouth-to-nose only Mouth-to-mouth without nose pinched Depth of compression in adults during CPR 1½ – 2 inches 2½ – 3 inches 1 – 1½ inches ½ – 1 inch Depth of compression in Children during CPR 1½ – 2 inches 2½ – 3 inches One-half to one-third depth of chest ½ – 1 CM Depth of compression in neonates during CPR 1½ – 2 inches 2½ – 3 inches ½ – 1 CM One-half to one-third depth of chest Rate of chest compression in adult and Children during CPR 100 / min 120 / min 80 / min 70 / min Ratio of CPR, single rescuer in adult is 15:2 5:1 30:2 15:1 In a new born the chest compression and ventilation ratio is 15:2 5:1 30:2 3:1 What does abbreviation AED stands for? Automated External Defibrillator Automated Electrical Defibrillator Advanced Electrical Defibrillator Advanced External Defibrillator What does abbreviation EMS stands for? Effective Medical Services Emergency Management Services Emergency Medical Services External Medical Support If you and your friend are having food in a canteen and suddenly your friend starts expressing symptoms of choking, what will be your first response? Give abdominal thrusts Give chest compression Confirm foreign body aspiration by talking to him Give back blows You are witnessing an infant who suddenly started choking while he was playing with the toy, you have confirmed that he is unable to cry (or) cough, what will be your first response? Start CPR immediately Try to remove the suspected foreign body by blind finger sweeping technique Back blows and chest compression of five cycles each then open the mouth and remove foreign body only when it is seen Give water to the infant You are witnessing an adult unresponsive victim who has been submerged in fresh water and just removed from it. He has spontaneous breathing, but he is unresponsive. What is the first step? CPR for two minutes and inform EMS CPR for one minute and inform EMS Compress the abdomen to remove the water Keep him in recovery position You noticed that your colleague has suddenly developed slurring of speech and weakness of right upper limb. Which one of the following can be done? Offer him some drinks, probably hypoglycemia Possibly stroke, get him to the nearest clinic Possibly stroke, he may require thrombolysis and hence activate emergency medical services May be due to sleep deprivation, make him sleep. A 50-year-old gentleman with retrosternal chest discomfort, profuse sweating and vomiting. What is next? Probably myocardial infarction, hence activates EMS, give an aspirin tablet and allow him to rest Probably acid peptic disease, give antacid and Ranitidine Probably indigestion, hence give soda Take him by walk to the nearest clinic.

After excluding the incomplete response forms the data was analysed on 1,054 responders. Permission was taken from all the institutional heads before involving the students and staff of their institution. The results were analysed using an answer, key prepared from the advanced cardiac life support manual [Table 2].

**Table 2:** Key answers

1 (b)	6 (d)	11 (a)	16 (c)
2 (c)	7 (b)	12 (c)	17 (c)
3 (b)	8 (c)	13 (d)	18 (d)
4 (c)	9 (c)	14 (a)	19 (c)
5 (a)	10 (d)	15 (c)	20 (a)

## RESULTS

**Figure 1:**
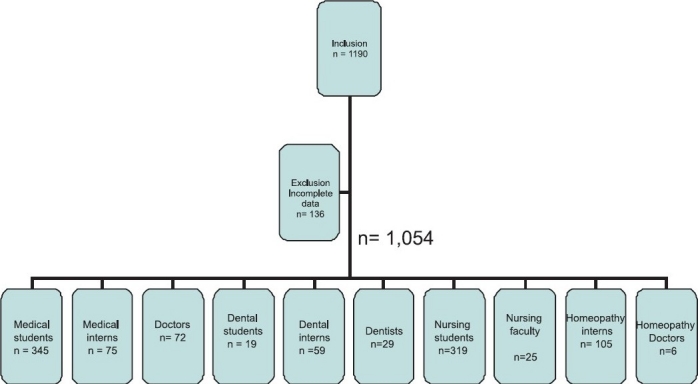
Over view of the responders

One thousand one hundred and ninety responders were included and 136 were excluded, as the forms they had filled were incomplete. Out of 1,054 responders 345 were medical students, 75 were medical interns, 19 were dental students, 59 were dental interns, 105 were homeopathy interns, 319 were nursing students, 72 were doctors, 29 were dentists, 25 were nursing faculty and six were homeopathy doctors [Figure 1].

**Figure 2:**
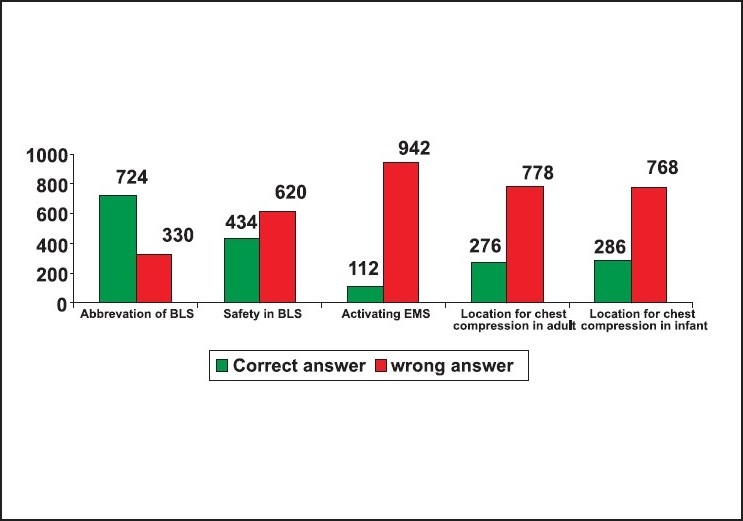
Response for the 1^st^ to 5^th^ questions n = 1054

**Figure 3:**
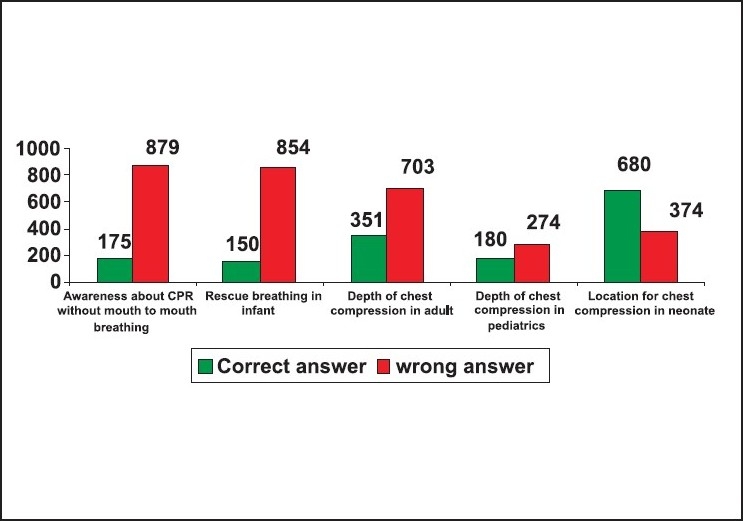
Response for the 6^th^ to 10^th^ questions n = 1054

**Figure 4:**
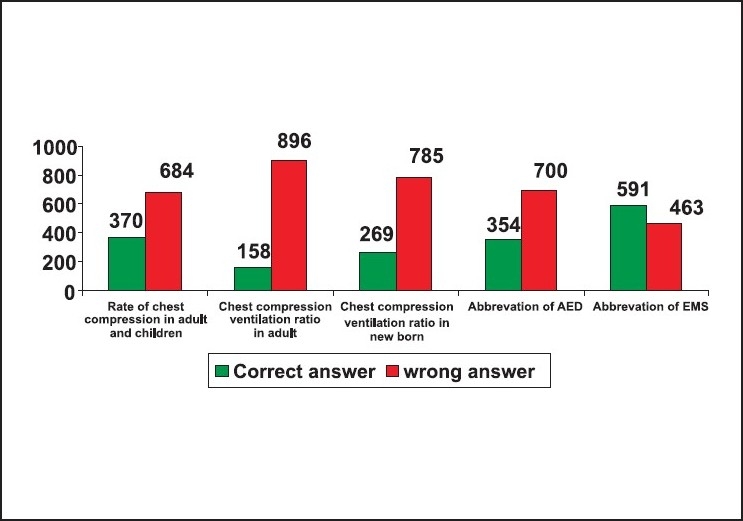
Response for the 11^th^ to 15^th^ questions n = 1054

**Figure 5:**
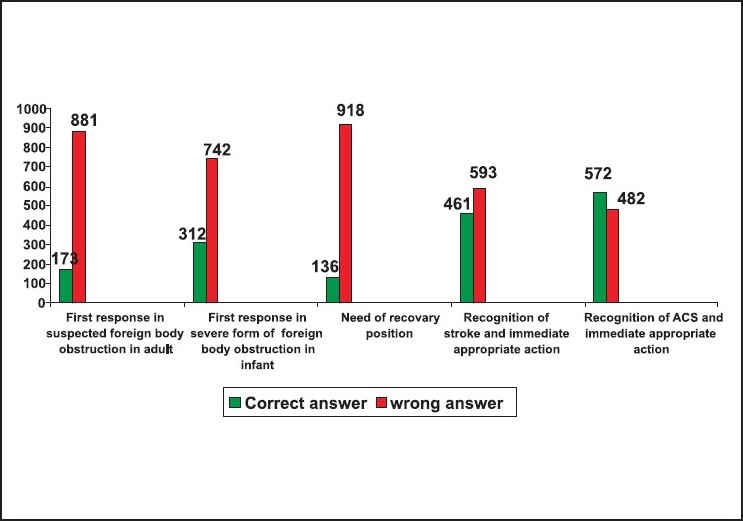
Response for the 16^th^ to 20^th^ questions n = 1054

Thirty-one per cent of the responders did not know the abbreviation of BLS as Basic life support. Fifty-nine failed to insist on looking for safety as the first step in BLS. Eighty-nine per cent failed to insist on activating EMS immediately after confirming the unresponsiveness in an adult. Seventy-four per cent did not know that the right location of chest compression was the mid chest. Seventy-three per cent of the responders did not know that the correct location of chest compression in an infant was one finger breadth just below the nipple line. Eighty-three per cent of the responders did not know alternative techniques of resuscitation when mouth-to-mouth ventilation was not opted. Eighty-four per cent of the responders failed to select mouth-to-mouth and nose technique as the rescue breathing for infants. Sixty-seven per cent did not know that the depth of chest compression in an adult was 1.5 to 2 inches. Eighty-three per cent did not know that the depth of chest compression in a child was one-third to one-half the depth of the chest. Thirty-five per cent did not know that the chest compression in an infant was one-third to one-half the depth of the chest. Only thirty-five per cent of the responders answered the rate of chest compression as 100/minute in adults and children CPR. Only fifteen per cent of the responders had correctly answered that the compression ventilation ratio in a child and adult single rescuer CPR was 30:2. Only twenty-six per cent knew that the ratio of compression ventilation in a new born was 3:1. Sixty-six per cent of the responders did not know that the abbreviation of AED was 'automated external defibrillator', and only fifty-six per cent knew that the abbreviation of EMS was 'Emergency Medical Service'. Eighty-four per cent did not know that the first step in helping a suspected foreign body obstruction victim is to confirm the severity of obstruction by talking to him. Only thirty per cent were aware about the right technique of foreign body removal from an infant. Only thirteen per cent knew about the role of the recovery position in a spontaneously breathing unresponsive victim. Sixty-six per cent of the responders did not know the early signs of stroke and only fifty-four per cent knew how to recognise and help a patient with acute coronary syndrome [Figures 2‐5].

**Figure 6:**
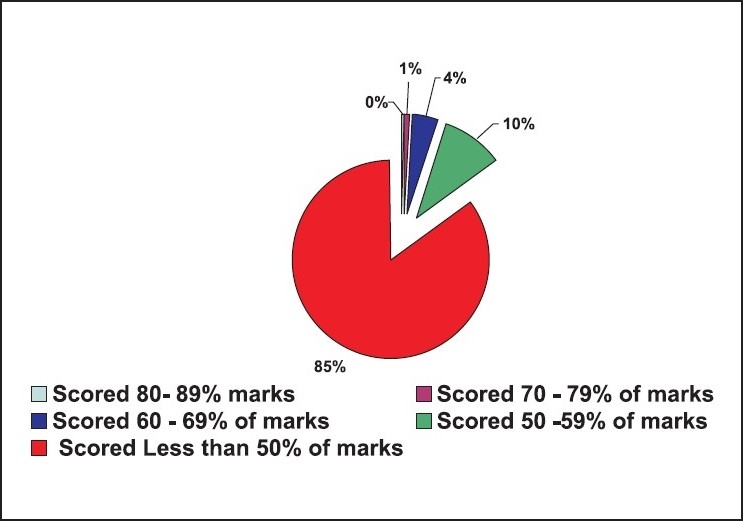
Responders and their scores n = 1054

No one had complete knowledge on BLS. Only two out of 1,054 (0.19%) had secured 80 – 89% marks, of these two, one was a medical intern and another was a preclinical teaching doctor. Ten out of 1,054 (0.95%) had secured 70 – 79% marks, of these, three were medical students, five were medical interns and two were pre-clinical teaching staff. Forty-three of 1,054 (4.08%) had secured 60 – 69% marks, of these, 12 were medical students, 11 were medical interns, six were pre- and para-clinical teaching staff, two were practising doctors and 12 belonged to the nursing faculty. One hundred and five of the 1,054 (9.96%) had secured 50 – 59% marks, of this 36 were medical students, 25 were medical interns, two were dental students, four were dental interns, five were nursing students, eight were pre- and para-clinical teaching doctors, five were practising doctors, another five were dentists, 13 belonged to the nursing faculty, one was from the homeopathy faculty, and another was an intern of homeopathy. The remaining 894 (84.82%) secured less than 50% marks [Figure 6].

Looking closely at the individual groups 83% of the medical students, 65% of the medical interns, 89% of the dental students, 93% of the dental interns, 99% of the homeopathy interns, 98.4% of the nursing students, 69% of pre- and para-clinical doctors, 59% of the practising doctors, 83% of the dentists and 83% of the homeopathy doctors had scored less than 50% of the marks [Figure 7a‐c.]

**Figure 7a:**
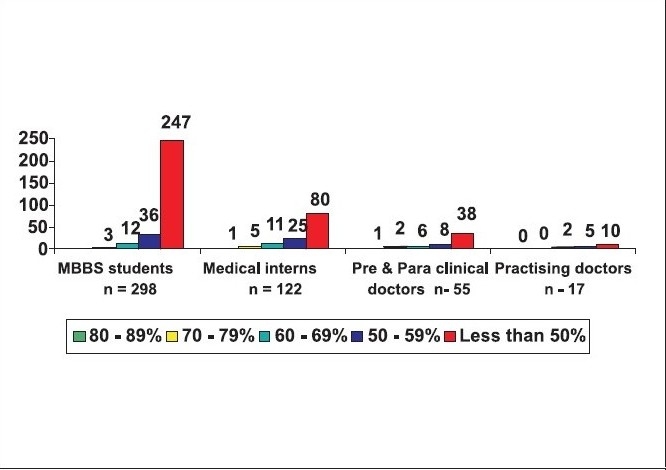
Awareness of BLS in medical college

**Figure 7b:**
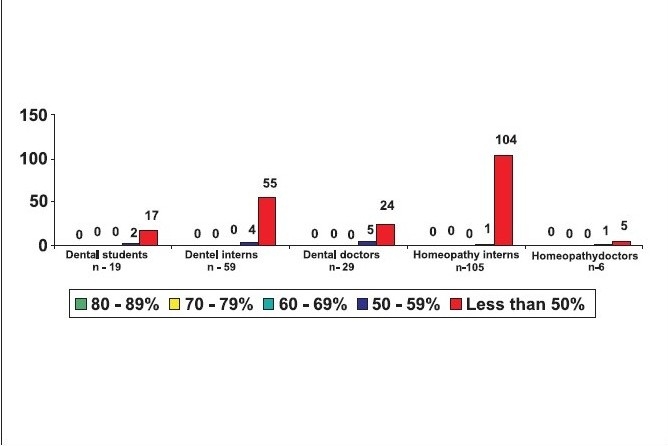
Awareness of BLS in dental and homeopathy field

**Figure 7c:**
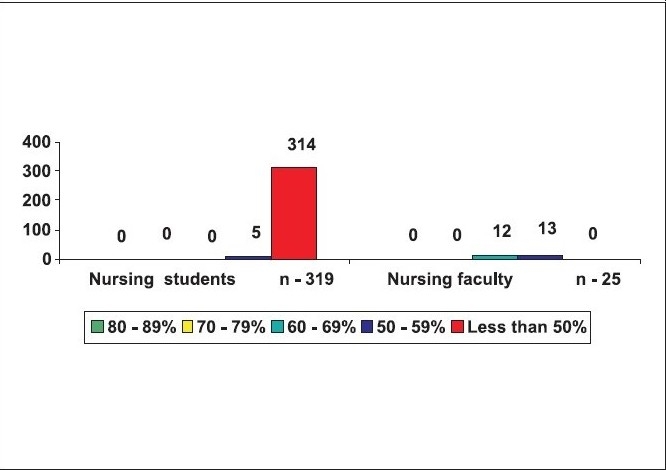
Awareness of BLS in nursing college

## DISCUSSION

The study results showed that medical, dental and nursing students and faculty in the study group were severely lacking in the awareness of BLS. Awareness of BLS was very poor in all the students. This study emphasized the cognitive approach to the general perception and skills of Basic Life Support, early recognition of stroke and acute coronary syndrome. The practising and teaching doctors in this study scored less compared to the nursing teaching faculty, this explained why many doctors were not good in carrying out effective CPR.[2,3] In the real sense many practising doctors, who were the teaching staff for the medical students did not come forward to respond to the questionnaire. It is now essential to standardise training in advanced life support and make it a mandatory component of all medical, nursing and para-medical school undergraduate curricula.[4] It is also equally important that teachers, school children, public and all lay persons from the community be taught the facts of basic life support and first aid.

The awareness on emergency medicine is increasing and The Medical Council of India has already approved emergency medicine as a separate specialty. Spreading awareness and teaching the basics of advanced life support to the medical and paramedical team as well as teaching BLS and first aid to the community will be the prime responsibility of this new emergency specialty.

### Limitations

Practical skills of basic life support could not be assessed in this study. Awareness on BLS among practising doctors was not satisfactory as a majority of them did not come forward to respond to the questionnaire.

### The need

To conduct BLS programmes in almost all corners and sectors of our society, with the intention of creating numerous basic life support responders.

## CONCLUSION

Awareness of Basic Life Support (BLS) among students, doctors and nurses of medical, dental, homeopathy and nursing colleges is very poor and needs to be improved.
